# To be at the right place at the right time

**DOI:** 10.1186/1746-1596-6-68

**Published:** 2011-07-22

**Authors:** Klaus Kayser, Stephan Borkenfeld, Torsten Goldmann, Gian Kayser

**Affiliations:** 1 Institute of Pathology, Charite, Chariteplatz 1, D-10118 Berlin, Germany; 2 International Academy of Telepathology, Heidelberg, Germany; 3 Clinical and Experimental Pathology, Research Center Borstel, Borstel, Germany; 4 Institute for Pathology, University of Freiburg, Freiburg, Germany

**Keywords:** Neighborhood interaction, order of structures, event, surface, entropy

## Abstract

**Aim:**

To analyze the hypothesis of events or neighborhood interactions that is based upon recognizable structures of systems which possess a surface in a four dimensional space - time constellation {x, y, z, t}. To include the theory of hierarchic order of structures and aspects of thermodynamically open systems, especially entropy, structural entropy and entropy flow.

**Hypothesis:**

Any structure is a space - time constellation that occupies a unique space in its environment. The environment can be a system too, and is assumed to be (nearly) constant. Structures can interact in their environment and create a new structure at a higher order level. Interacting structures that create a surface are called a system. Starting from the bottom, such a system is characterized by its inner structures, its surface function, and its neighborhood. Interaction with a neighboring system is called an event. An event can alter a system, create new systems or induce the decay of a system, dependent upon the surrounding lower level system (background).

**Results:**

The hypothesis results in a uniform theory about matter, life, diseases, or behavior. Concrete applications permit the estimation of duration of life in man, for example the effect of solid cancer in man, or appearance of protozoans in sexual or asexual reduplication. In addition, it can successfully describe the development of the universe (small exceed of matter above antimatter at the big bang), or the increase of structures (and systems) with increasing time (development of intelligent systems). The three dimensional space possesses the lowest number of mandatory dimensions to implement such a system.

## Introduction

The interpretation of our environment and the search for the "final cause" or destination of our individual life is assumed to be a question of belief that is out of the range of natural sciences [1-3] Never the less we are confronted with convenient or displeasing events in our daily life, which at least some of us would like to be explained [1,3]. Why could I escape the imminent accident? Why did I become agitated in this discussion despite I am personally not involved in any of the disagreed issues? Or, more difficult, what are the reasons that I do or do not understand some properties of my environment, for example those that are described by Einstein's general theory of relativity? Why was Elvis Presley that successful when he started his career? How can I reach the same level of success?

Many of us would answer: You have to be at the right place at the right time, or to avoid the wrong place at the wrong time! Try not to understand what you cannot understand either!

This article introduces a hypothesis on right time and right place, which tries to generalize our daily experiences of right (or wrong) place and right (or wrong) time.

## Theoretical considerations

Obviously, man belongs to a specific class of creatures which all are characterized by certain features and have to obey general laws of life. These include a very early stage of the individual that starts with fertilization, followed by growth and maturation, and finally ends with its death. In addition, specific functions develop such as assurance of energy supply (nutrition), or information exchange with the environment, with individuals of other or the identical species, and, at the highest level, the recognition of itself (awareness).

Basically, every individual can be distinguished from its environment, i.e., has to possess a surface [4,5]. The surface is of a lower dimension and divides the individual's space - time constellation into two non-overlapping spaces: the "enclosed" space which belongs to the individual, and the "outer one" which we usually call environment. The surface assures the energy supply to stay alive, to exchange information and to communicate. It possesses different properties at different areas. Thus, from the thermodynamic point of view, the system is "open" [6,7].

We can distinguish between two different "classes" of individuals, those that are free to move in the space - time constellation (animals), and those that are not (plants). Interestingly, plants possess a less "complicated" surface compared to that of animals. Plants can live with roots, boles, branches, leaves, and blossoms already. The surface of animals includes skin, nails, intestines, air spaces, ears, eyes, or hair, which are again constructed in organ specific manner. For example, the air spaces are differentiated into the nose, trachea - bronchi, terminal bronchi and air exchange spaces (alveoli). They possess different structures and different functions [8].

In addition to the described living systems there exist other systems that also possess a "surface", which, however, cannot reduplicate in general and seem to be "stable". These non-living systems (stones, metals, cars, computers, etc.) can exchange energy and possess nested internal structures and systems too and might become close to those that we call life.

We can now formulate a "basic law" derived from our experience:

All systems of interest are embedded in a metric space. In our environment it is a four dimensional space, that can be separated into three non-directed coordinates (x, y, z), and into a directed one (time, t). (x, y, z) and (t) have not to be necessarily independent from each other, in fact, they are not [9].

Every structure and the related system exist in its own space- time environment. In other words, systems do not overlap. Either the space or the time has to differ for each system.

From this observation we can derive a "constellation" of systems, i.e. a neighborhood. Two different systems can be neighbors, either in space, or in time, or in both. How can we analyze the constellation?

The first question to be answered is: Are the systems neighbors, or not? Secondly: What are the conditions to be neighbors?

For practical reasons, and usually limited to analysis of visual information, the most frequently applied neighborhood condition is based upon Voronoi's or Dirichlet's tessellation [10]. The graph theory is the preferred mathematical approach to derive information from spatial constellations (structures) [11,12].

Using an appropriate neighborhood condition and formulations of graph theory (for example the minimum spanning tree and derivatives) we can analyze conditions and consecutives of interaction between neighboring systems [13-15]. They can be derived from observations in living systems, and hopefully can serve for theoretical approach to describe the basic principle of the universe.

To start with: In our daily life, we all are involved or observe numerous constellations, which we call "events", such as fall in love, good luck, accident, acute heart failure, etc. What about our universe? We explore explosions (novas), new stars, dying stars, etc. In addition, the most frequently accepted theory of the universe's origin is the theory of the big bang [16]. Although not clearly defined at this stage, we can call the observations and the big bang an "event". Do all these events have something in common, or is it just an "accidental" expression of occurrences that differ "in principle"?

### What is an event?

In our environment, we can define an event as the result of at least two different time - space constellations of (living) systems (structures of the space-time constellation) that fit together, and induce changes in either one or both of them. Examples are fertilization of an egg by a sperm, a lion kills a gazelle, two cars (drivers) crash, a band plays in front of the auditorium, a politician reads a news paper, etc.

If the constellations do not fit together (in other words, if they do not interact or exchange information), nothing happens (examples: an egg lies on a plate at breakfast, the gazelle escapes the lion, the seed cannot sprout in the earth due to lack of water, one driver can avoid the accident, the politician does not understand the newspaper, etc.). The interaction can affect all involved systems, the environment only, or only one of the systems. The interaction is usually triggered by spatial movement of one system (lion jumps on a gazelle), by inner changes of one (or of both) structures, (examples: sperm enters an egg), interaction of additional systems (additional lions eat from the flesh), or by changes of the environment (sprout of a seed after rain). Thus, we have a constellation that includes a minimum of two partners. One of them is a system that can be described by its location in the space - time environment, its surface, its inner structures, and interactions that have to involve the surface.

### What are the descriptors of an event?

Every living system occupies a certain limited space for a limited time. Its boundary is called surface. Its inner space commonly includes additional limited spaces for the same or a smaller time the system is alive. In other words, the system itself possesses inner structures which are often arranged in a hierarchical order, or nested. In life, they are often arranged as a functional unit (organ, vessel, nerve, cell, nucleus, etc.), which means they act as systems that are embedded in the inner space of the whole (higher order) system.

An example is: Heart beats in a running person, the cardiac vessels contain blood, this blood flows through the heart vessels, erythrocytes are transported with the blood stream. The runner is the "environment of the heart, the heart the environment of the intra-cardiac vessels, the vessel the environment of the blood, and the blood that of the erythrocytes. The inner space can be considered as an "inner environment" that obviously can act in a similar manner as the environment of the whole system itself.

Dependent upon the basic level that we are free to choose, the result will be a hierarchical order of structures and systems as described by Kayser et al. [17-19]. These authors started from the light microscopic level (cells) and could demonstrate the biological significance of system surface and constellation parameters in solid cancers, i.e. on the prognosis of the patients [4,14,18,20,21].

These considerations postulate a constant space - time environment that we can use to describe structures. It serves for the coordinate system which defines the neighborhood condition and derived parameters such as structural entropy [4].

In a first step we limit our considerations to stable basic living systems, such as man, bird, snake, dog, etc. Stability means, that at least one system of the involved systems does not (significantly) change its inner systems, structures or surface prior to the interaction with another system or the environment.

According to our postulate and experiences the interaction requires that the involved systems are spatially separated from each other. The interaction will only take place if all involved partners fulfill a neighborhood condition that triggers the interaction.

We can now take a look at the neighborhood conditions and postulate:

1. The occurrence of an interaction is called an event.

2. Obviously events take place in the environment of the involved systems.

3. An environment is a scalable space - time constellation with constant features (air, water, space, etc.) {r, t, f(r, t)}. Its range is large in relation to the expansion of the involved systems, as long as the environment can be considered as a system too. Thus, we can neglect the outer surfaces of the environment, and it is irrelevant whether they exist or not.

4. Systems occupy space and time {rs, ts, fs} of the environment {r, t}, with rs c r, ts c t.

5. If systems dissolute, they are replaced by different systems or by the environment.

6. The constellation of the systems within their environment defines the neighborhood condition.

7. A spatial and time defined neighborhood is mandatory; however not sufficient for interaction.

8. The release of an interaction requires additional constellations of the involved systems (hunger of the lion, intelligence of the politician, properties of the surfaces in egg - sperm interaction, etc.).

9. The environment and the structures obey the same basic laws (physics, emotional, biological, etc.).

### Derivatives

#### Structural entropy

A frequently used approach to analyze spatial distributed systems is the concept of entropy [22,23]. The concept of entropy can be interpreted as a measure of "uncertainty" or as a measure of order in a macro-system with several microstates [4].

When we consider a constellation (environment) that is composed of numerous distinct properties (systems) *i *each being measured with a probability *pi*, the distance from the constellation's end stage can be computed to

(K = constant, so-called Boltzmann constant), with the condition ∑(p*_i _*)= 1.

The entropy S measures the "distance of the systems" from "predictable end stage" as long as no interaction occurs. In other words, if the probability *pi *is only related to the total number N of observable systems or (micro) states (for example 6 in case of a usual die), then *p_i _= N_i_/N *holds, and we will obtain the so - called Boltzmann - Gibbs distribution of *pi*. The entropy *S *is maximum if the *pi *do not differ, i.e., once the system has reached its end stage.

We can now consider the systems that interact or count "events". The entropies can then be simply added (which makes them unique), if we assume no dependencies (strong chaos or the so-called Boltzmann - Gibbs distribution); however, it might become more complicated for dependent systems (weak chaos). In addition, we might take into account regularities (symmetries) that can frequently be noted in nature. Details and conditions of this entropy have been discussed by Tsallis [22,23].

Examples with clinical applications have been reported [4,18,22,24]. Starting from the level of individual cells, any healthy tissue possesses regular, i.e. repeating structures that can be aggregated to new (higher order) structures. Diseases (for example cancer) disturb these symmetries by more or less spatially random growth. Assuming additive entropies and features that are derived from "normal field forces" we get the following result:

Δm_ik _= micro- stage differences between neighboring cells (distance, mass, staining intensities, etc.);

mm_k_, Δm_k _= mean of micro- stages in the macro- stage k.

The more general formula to calculate the "distance of living systems" from the end stage is

Δfe_ik _= micro- stage differences between an event and those of non-involved systems (distance, hunger, sex, age, velocity, mass,, etc.);

ft_k_, = mean of micro- stages in the system k out of the neighborhood;

Δfe_k _= difference of microstages between an event k and the systems not involved.

#### Flow of entropy

Until now we have applied the concept of entropy to so - called closed systems. Closed systems have a fixed and impenetrable surface, and their development solely depends upon their embedded macro- and microsystems, i.e., structures, events, classes, and the internal relationships. These systems only crudely reflect to biology systems, which require an exchange of energy and other features to stay alive. In addition they develop in a fixed direction of time, and considerations derived from the field of irreversible thermodynamics seem to be more appropriate [6,7].

Prigigone analyzed the development of open thermodynamic systems and introduced a dynamic derivative of the entropy, the so - called flow of entropy [25,26]. According to the theorem of Prigogine (1959) an open thermodynamic system tries to minimize the creation of new entropy and to become indistinguishable from its environment in such a manner that d/dt(T*dS/dt) = >0 holds true for the final stationary stage (*T *= temperature) [6,7,26]. Eigen demonstrated in accordance to Prigigone's theorem that major changes in any open biological system can only occur in stages of large "local" entropy differences in comparison to the individual environment [27].

In higher order biological systems the flow of entropy can be calculated in circumscribed diseases such as cancer [24,28,29], or in "agglutinated" populations with events. If we consider events (for example dividing cells) as the main production of heat in a circumscribed system, and that the produced heat can be only transported into the (healthy) environment via the internal and outer boundaries, the corresponding formula of the entropy flow *EF *[24,28] can be derived to:

*EF*: entropy flow;

*E(MST) *: structural entropy;

*Sph*: numerical fraction of heat production (events, proliferating (tumor) cells, etc.);

*OS*: outer surface;

*IS*: inner surface.

#### Development in biology

Living biological systems are characterized by (nearly) constant structures and inner systems, and structure/system - associated functions. Biological functions include static and dynamic flows of energy, heat, molecules, and electromagnetic fields. They ensure either a sufficient amount of free energy embedded in an appropriate environment, or an adequate information transfer. Both procedures are essential to keep the system alive:

Obviously, systems exist that have lost their functions. We call such systems a corpse. The opposite, a system that has lost its structure and still possesses its functions is known by children and called a ghost (and would not be an appropriate object of natural sciences in general).

Thus, structures are essential to maintain a constant and low entropy level within the living system, and have to steer the flow of entropy [4,5,27]. They also require free energy in order to compensate their own entropy increase.

To further analyze the entropy in living organisms, the following theorems are stated:

The functions of a living system are a mixture of export and import of its own products and that of the environment. The import/export machine is regulated by specific recognition processes which are part of the neighborhood condition. At cellular and subcellular (molecular biology) receptors, domains, antigens, etc. take this duty.

Assuming that a living system possesses defined spaces (macro- stages) of lower entropy compared with their environment a mechanism has to exist that ensures the entropy difference between the system and its environment. The only way such a machine can work is to use free energy to export the created heat of the living system. In addition, all involved products (molecules, energy, entropy) have to pass the system's surface. If we agree that this entropy diminishing machine obeys general biological laws, we have to take into account that it will become less efficient with increasing age. Its efficiency has to be renewed after certain times in order to become more efficient again (for example during embryogenesis or phylogenesis).

**Figure 1:**
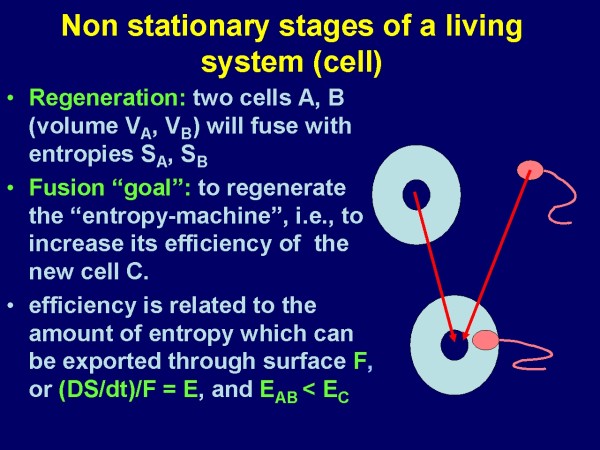
**Scheme of cellular fusion from the viewpoint of the entropy concept: The highest efficiency of entropy transportation is obtained, if the surface/volume fraction of the new generated cell becomes a maximum, which obviously requires V_A _>> V_B_**. [4].

**Figure 2:**
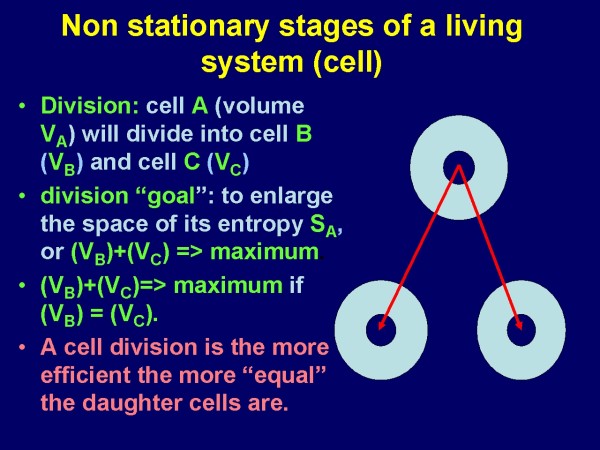
**Scheme of cell division from the viewpoint of the entropy concept: The more equal the daughter cells are, the greater is the generated space of lower entropy; i.e., V_A _= maximum if V_B _= V_C_**.[4].

More in detail, nature might use two different techniques to lower the entropy in a living system: a) to increase the efficiency of the machine itself, and b) to increase the space the machine is responsible for. At the cellular level both techniques can be observed, as illustrated in Figure 1 and 2, taken from [4]

These two examples demonstrate that living systems can be considered as complicated arrangements of nested structures and inner systems that form a surface and obey basic physical laws. For a certain space and time constellation they possess a constant arrangement. They tend to multiply if a suitable neighborhood condition exists (right space and right time). In addition, they derive from an environment, which is under this assumption another, lower level system. However, what about the environment (lowest level structure) or the universe, or what is assumed to exist below the "lowest level"?

### Space time considerations

From the early beginning man tried to develop ideas about the origin of the universe, and to formulate a unique formula that can serve for a general description [1,2]. Additional aims are to explain the existence of man, the obtained general natural laws, and future development of our species [1,2].

In doing this, man has detected, observes and measures systems and structures in the universe, for example constellations of stars, galaxies, etc.

However, does an "environment" of the universe exist? If yes, what is its relationship to the observed structures and their history? How does our hypothesis of right space and right time fit into such huge dimensions?

There are increasing indicators that black matter (energy) exists in the universe which we might consider as the environment of the universe [16]. Is this indication a chance that our hypothesis does not solely remain a fiction?

Assuming that a general (at the lowest level) positioned environment of the universe exists (which we call black matter), and that it possesses some similarities to the development of biological systems, we can try the following theorems:

1. We are living in a four-dimensional space - time constellation (x, y, z, t).

2. This constellation is the lowest level structure derived from the black matter.

3. It is scalable, and contains several ordered and nested structures, that finally form living systems after passing thru numerous higher order systems and structures.

We can then ask: What is the dimension of the black matter?

According to our hypothesis the universe itself is a structure. It might still contain black matter at those "spaces" that do not contain additional, higher order structures, however, our hypothesis predicts that the black matter is not present at other higher order structures, which include "occupied" spaces (mass, stars, radiation, etc).

The dimension of the black matter would be (r = 0, t = 0), as r > 0 and t > 0 are the boundaries of the derived lowest structure, the universe. From the viewpoint of entropy we could call this stage "condensed entropy" with undefined macrostages and an unlimited number of microstages. Once released, the microstages will organize themselves in macrostages, i.e. will form structures (and a surface). Their number as well as the temperature of the system will decrease with increasing {r,t}.

In this case our hypothesis is "very close" to the inflation theory of the big bang, which distinguishes early structures such as quarks, antiquarks, electrons, positrons, at later stages more complicated structures such as mesons, neutrons (they have already an internal structure), protons, finally atoms, molecules, etc. [30].

Dependent upon the structure and its neighboring structures the following events can occur:

1. A new (higher order) structure is created (with a new surface, interactions of quarks, electrons, etc).

2. The structure is dissolved (for example electron - positron interaction) and a lower order structure remains (string). If the dissolved structure is at the "lowest level", all structures are lost, i.e., the bottom of the "environment" remains, which is the stage at {r = 0, t = 0}.

3. Even if we assume an equal distribution between matter and antimatter, the creation of new structures is a statistical process and controlled by neighborhood conditions. Therefore, it is unlikely that structures of matter and of antimatter will appear in exactly the same number. Once the constellation (that of matter or that of antimatter) is able to create slightly more structures N (Nmatter > Nantimatter), immediately the effect will be gained and results in an overwhelming predominance of those structures that are only of little advantage in the beginning. Thus, we can describe the existence of matter.

Our hypothesis that surfaces, i.e., boundaries between different space - time constellations and events can serve for description of the involved structures and systems seems not to be a simple fiction. In addition, it possesses the following advantages:

1. We have only to describe the "differences" or derived "flows" between the involved systems, and have not to analyze the whole arrangement.

2. We can estimate the "age of the systems". Obviously those systems are "older" or "more differentiated" that possess more internal structures (nested systems).

3. We can apply the hypothesis to different sciences such as molecular biology, cellular societies, living organisms, societies, or basic theories in physics such as that of the big bang.

Our hypothesis requires that at least one of the space - time parameters has to be directed (otherwise no systems and correspondingly no surfaces will be created).

It explains the increase of "intelligence in living systems with increasing time" because more complicated systems (systems that contain more nested structures and inner systems) require more space and time to be created and, therefore, are older than less complicated ones. They have more interactions (information flows) between the internal structures and between the systems and their environments.

If (biological) systems multiply, stabilization (feedback) mechanisms have to exist between their structures (otherwise a hierarchy of structures could not be established). The nesting of inner systems and structures ensures the stability of the whole living system.

The measure entropy and derived parameters such as entropy flow can be specifically applied to forecast the length of systems' life [20,21,24,25]. Man's life occurs in a space of four dimensions. Could it exist in a space of lower dimensions?

#### Dimension of space

Our basic environment which we call universe can be described by a four dimensional space - time configuration [1,9]. The neighborhood condition postulated by our hypothesis requires that all derived structures have to occur in these four dimensions only, because they cannot create a boundary outside of this space. In addition each structure has to exceed a certain size because at least two neighboring basic elements are mandatory.

**Figure 3:**
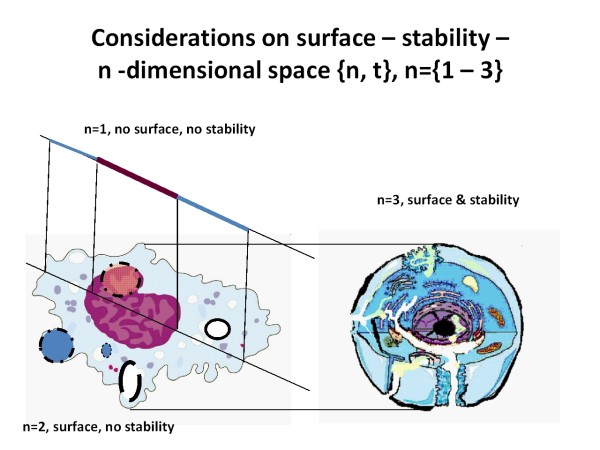
**Projection of a cell scheme (n={3,t} to a n={2,t} plane, and that of the n ={2,t} plane to a n={1,t} line**. Only a three dimensional space offers stable internal and external "open", i.e., constantly porous boundaries.

Simple calculations can give us some impression about the embedded structures and possible events of such systems in relation to the postulated number of dimensions:

If we assume (in addition to the time dimension) a one dimensional space {1,t}, the maximum number of configurations to create a structure or an event results in two neighbors only. Two - not necessarily identical - neighbors are mandatory and sufficient to form the first event, followed by another four neighbors (two events) at the corresponding start and end points. Internal structures cannot be built because such a system does not possess a boundary (or surface). The dimension of the boundary would be {0,t}, as surfaces require one dimension less compared to those of the original space {1,t}, (see Figure 3).

If we assume a two dimensional space {2,t}, the maximum number N_max _to create a first event results in nine neighbors, the minimum is two again. The more extensive the covered constellations are the higher is the mandatory number according to the formula (Square basic elements and neighborhood condition based upon connecting edges and vertices are postulated)

Internal structures can be built according to the formula

The boundary would be a one -dimensional line. Such a boundary cannot serve for mandatory interactions between systems or the system and its environment (for example entropy exchange), because the mandatory "breaks" cannot be stabilized (see Figure 3).

The next higher space, the three dimensional space {3,t} is that of the lowest number of dimensions that fulfill the conditions a) to possess a boundary (of the dimension {2,t}), and b) to create and stabilize "channels for interaction with the environment" (of the dimension {1,t}).

Thus, nature has chosen the lowest ever possible number of space dimensions to create systems that finally reach the level of living organisms including man, and to permit us to be at the right place at the right time.

## Competing interests

The authors declare that they have no competing interests.

## Authors' contributions

NA, All authors read and approved the final manuscript

## References

[B1] Einstein A Albert Einstein The human Side - New Glimpses from his Archives 1979 Princeton, New Yersey: Princeton University Press

[B2] Heisenberg W Wandlungen in den Grundlagen der Naturwissenschaft 1959 Stuttgart: S. Hirzel Verlag

[B3] Kayser K Stauch G Zeitgedanken und Spiegeldenken 2000 Baden - Baden: Rendevous Verlag

[B4] Kayser K Kayser G Metze K The concept of structural entropy in tissue-based diagnosis Anal Quant Cytol Histol 2007 29 5 296 308 17987810

[B5] Trintscher KS Biologie und Information - Eine Diskussion über Probleme der biologischen Thermodynamik 1967 Leipzig: B. G. Teubner Verlagsgesellschaft

[B6] De Groot SR Thermodynamik irreversibler Prozesse 1960 18/18A Mannheim: Bibliographisches Institut

[B7] De Groot SR Mazur P Non-equilibrium Thermodynamics 1962 Amsterdam: North-Holland Publishing Company

[B8] Kayser K Analytical Lung Pathology 1992 Heidelberg, New York: Springer

[B9] Einstein A The Meaning of Relativity 1956 Princeton, New Yersey: Princeton University Press

[B10] Voronoi G Nouvelles applications des parametres continus a la theorie des formes quadratiques, deuxieme memoire: recherches sur les paralleloedres primitifs J Reine Angew Math 1902 134 188 287

[B11] O'Callaghan JF An alternative definition for neighborhood of a point IEEE Trans Comput 1875 24 1121 1125

[B12] Zahn CT Graph-theoretical methods for detecting and describing Gestalt clusters IEEE Trans Comput 1971 C-20 68 86

[B13] Kayser K Theory of sampling and its application in tissue based diagnosis Diagn Pathol 2009 4 6 10.1186/1746-1596-4-6 19220904 PMC2649041

[B14] Kayser K Neighborhood analysis of low magnification structures (glands) in healthy, adenomatous, and carcinomatous colon mucosa Pathol Res Pract 1986 181 2 153 8 3737471 10.1016/s0344-0338(86)80004-8

[B15] Ruelle D Zufall und Chaos 1993 Berlin, Heidelberg, New York: Springer

[B16] Cheng TP Relativity, Gravitation and Cosmology: a Basic Introduction 2005 Oxford, New York: Oxford University Press

[B17] Kayser K T.P. G. Ferraté, A. Sanfeliu, H. Bunke Application of structural pattern recognition in histopathology Syntactic and structural pattern recognition 1988 Springer: Berlin Heidelberg New York 115 135

[B18] Kayser K TNM stage, immunohistology, syntactic structure analysis and survival in patients with small cell anaplastic carcinoma of the lung J Cancer Res Clin Oncol 1987 113 5 473 l80 10.1007/BF00390042 3040767 PMC12248402

[B19] Kayser K Hoeffgen H Pattern recognition in histopathology by orders of textures Med Inform (Lond) 1984 9 1 55 9 10.3109/14639238409010938 6717163

[B20] Kayser K Combined morphometrical and syntactic structure analysis as tools for histomorphological insight into human lung carcinoma growth Anal Cell Pathol 1990 2 3 167 78 2177350

[B21] Kayser K Primary colorectal carcinomas and their intrapulmonary metastases: clinical, glyco-, immuno- and lectin histochemical, nuclear and syntactic structure analysis with emphasis on correlation with period of occurrence of metastases and survival Apmis 2002 110 6 435 46 10.1034/j.1600-0463.2002.100601.x 12193204

[B22] Gell-Mann M Tsallis C eds Nonextensive Entropy: Interdisciplinary Applications 2004 Oxford University Press: Oxford

[B23] Tsallis C Introduction to Nonextensive Statistical Mechanics 2010 Berlin: Springer

[B24] Kayser K Gabius HJ The application of thermodynamic principles to histochemical and morphometric tissue research: principles and practical outline with focus on the glycosciences Cell Tissue Res 1999 296 3 443 55 10.1007/s004410051305 10370131

[B25] Kayser K Molnar B Weinstein RS Virtual microscopy - Fundamentals, Applications, Perspectives of electronic tissue - based diagnosis 2007 Berlin: VSV Interdisciplinary Medical Publishing

[B26] Prigogine I Introduction to Thermodynamics of Irreversible Processes 1961 2 New Yorck: John Wiley & Sons Inc

[B27] Eigen M Selforganization of matter and the evolution of biological macromolecules Naturwissenschaften 1971 58 465 523 10.1007/BF00623322 4942363

[B28] Kayser K Gabius HJ W. Graumann Graph Theory and the Entropy Concept in Histochemistry Progress in Histochemistry and Cytochemistry 1997 32 Stuttgart, Jena, Lübeck, Ulm: Gustav Fischer 9551486

[B29] Kayser K Towards an automated virtual slide screening: theoretical considerations and practical experiences of automated tissue-based virtual diagnosis to be implemented in the Internet Diagn Pathol 2006 1 10 16764733 10.1186/1746-1596-1-10 PMC1524814

[B30] Guth A Eternal inflation and its implications J Phys 2007 A40 6811 6826

